# Exploring Determinants of Population Ageing in Northeast China: From a Socio-Economic Perspective

**DOI:** 10.3390/ijerph16214265

**Published:** 2019-11-02

**Authors:** Ling Yang, Kai Zhao, Zhen Fan

**Affiliations:** 1 School of Economics and Finance, Xi’an Jiaotong University, Shaanxi 710061, China; lyang@xjtu.edu.cn; 2 School of Economics, Renmin University of China, Beijing 100872, China

**Keywords:** ageing, socio-economic factors, birth rate, northeast China

## Abstract

The paper aims to examine the population ageing process in northeast China, typically perceived as a region experiencing dramatic demographic change and socio-economic slowdown that is much deeper and more significant compared to other regions. Using the 2000 and 2010 census data at the sub-regional level, the SEM (spatial error model) estimation suggests that at least seven socio-economic factors are associated with the evolution of the ageing pattern in northeast China, including birth rate, mortality, education, healthcare conditions, the level of economic development, urbanization, and population mobility. However, these associations vary according to time and space, which are further confirmed by the geographical weighted regression (GWR). These findings imply that there are complicated and diversified factors which may be associated with the deteriorating population ageing at the local level in northeast China. Therefore, the sustainable development of the northeast region may not be delivered by dichotomous policy interventions, such as the control of birth rate or mortality rate, as many of the previous studies have focused on; instead, the implementation of ageing policy shall be consistent and complementary with the principles of social benefits, for example, providing incentives for improving regional economic structures, or by policies aimed at building up an adequate “tolerant culture” for slowing down population outflows.

## 1. Introduction

Population ageing, among all other effects brought by demographic changes, has become a primary focus that attracts academics and policy markers’ attention. Even though studies have shown that population ageing impacts on every aspect of socio-economic development, ageing has not received enough attention in economic and population research discourse in China until the beginning of the 21st century. Previous studies have either focused on exploring the mechanistic relationship between the presence of ageing and the consequences it caused, such as growing disparities between different regions and misallocation of healthcare resources, or the counter-measures that could alleviate the negative effect of ageism. However, this strand of inquiry, to a certain degree, only proposes “one-size-fits-all” solutions, while it lacks recognition with regard to: (1) the dynamic trajectory of ageing at the sub-regional levels in China; (2) the differential effects of socio-economic factors on ageing across different regions, and; (3) the spatial neighbor effects on ageing between regions and cities. As a result, great differences in aims, stipulations, implementations, and context of regional ageing policies have not been considered, and not surprisingly, it remains unclear how the microscopic strategy and integration measures can be dynamically formulated in complicated socio-economic scenarios.

In addressing these issues, the present study adopts comprehensive spatial models to understand the function and transmission mechanism of ageing at the sub-regional level in China. We choose northeast China as the prime example of spatial unit, as it is experiencing dramatic demographic change and socio-economic slowdown that are much deeper and more significant compared to other regions in China. Using global/local spatial autocorrelation and geographical weighted (GWR) models, the effects of various socio-economic and demographic factors, such as birth rate, illiteracy rate, medical and health provision, mortality, industrial structure, urbanization level, and population mobility on ageing were simultaneously examined, and we sought appropriate answers to the following questions: (1) from a spatial–temporal perspective, what is the ageing situation in northeast China over the last two decades; (2) what are the most important determinants that lead to this ageing dilemma. The findings suggest that the situation of population ageing aggravates over the period 2000–2010 in northeast China, and this phenomenon is particularly prominent in developed areas such as Liaoning province. However, this ageing pattern is time-varying, as birth rate, mortality, education, and healthcare conditions were more likely to influence the situation of population ageing in 2000, which were further replaced by industrial sector size, urbanization, and population mobility in 2010. Therefore, this study provides meaningful references for policy makers to understand how socio-economic development affects population ageing.

The remainder of this paper is structured as follows. Section 2 reviews relevant historical context and theories. Section 3 introduces the methodology employed in this study including data collection methods, the research model and variable definitions. Section 4 shows the analysis of results. Section 5 and Section 6 concludes the study with discussions of recommended strategies for further strengthening the ageing countermeasure in northeast China, limitations of the current study, and possible directions for future research.

## 2. Historical Context and Literature Review

**Figure 1:**
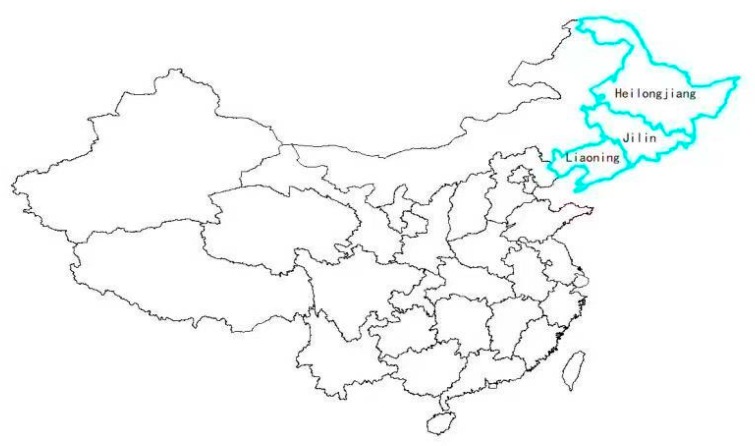
The location of the northeast region in China.

Northeast China (i.e., Liaoning Province, Jilin Province, and Heilongjiang Province, see Figure 1), as the first and one of the most important industrial bases, has made significant contributions to the overall economic development of China over the past 40 years. However, since the policy of the reform and opening up to the world were carried out, its economic development and urban construction have gradually dropped behind the east coastal areas. With such an economic stagflation, the northeast region appears to undergo a deep socio-economic transformation, most notably the rapid change of population structure. According to “Report on the Development of China’s Floating Population 2016”, the northeast region is facing great population crisis issues, such as extremely low fertility, population ageing acceleration, and unbalanced age structure. Compared to other provinces and municipalities in China, the total fertility rate in the northeast region is only 0.75, which is much lower than that of the national average (1.18). The population growth has also stalled, which was only 0.21% on average over the period 2006–2015, less than half of the national average (0.5%). These processes have been accompanied with a significant increase of the media age of the population. In 2015, the median age of the northeast population was 43 years, whereas that of the national population was only 38 years. Moreover, population loss in northeast China is also significantly observable. Based on the census data, the net outflow of population in 2010 was about 2.19 million, which was five times more compared to the figure in 2000, further aggravating the population ageing.

A number of studies have demonstrated the strong linkage between population ageing in China and various demographic factors such as birth rate and mortality rate [1,2,3]. These factors are considered as the dominant factors affecting the population ageing, which are consistent with the international research. For instance, Alders et al. (2005) [4] showed that population growth and changes in living standards and quality all have impacts on the age structure of the population, which eventually leads to an ageing population. Bloom et al. (2015) [5] pointed out that population ageing is mainly caused by the extension of average life expectancy, ageing, and fertility of “baby boomers”. In comparison, some experts have proffered alternative explanations. Observing the population flow trend in Fujian province, where it is usually acknowledged as a region of emigration, Zhou et al. (2016) [6] highlighted the importance of emigration as Goldstein et al. (2009) [7] suggested, by arguing that the general population outflow has a stronger effect in promoting population ageing. This strand of research, rather than emphasizing the role of anthropological factors, has extended the discussion of population ageing to the context of education [8,9], urbanization [10,11], medical conditions [12], and economic development [13,14]. Yet, despite the efforts to include a range of factors reflecting the complexity associated with ageing issues, there is still room for further advancement, especially the ‘spatial-context’ dimension of this concept. Some research has suggested that understanding the reason and impact of population ageing not only needs to consider the interdependence of population-society/environment interactions, but also the spatial effect on this base [15,16,17,18,19,20,21]. In order to explore the determinants of ageing in Jiangsu province over the period 2000–2010, Yang et al. (2016) [22] applied the spatial error model, and the results indicated a time-varying effect of economic development on ageing. Extending a similar research discourse, Zhao et al. (2012) [23] demonstrated that the population ageing was mainly affected by the inertia of population growth and its spillover effects among regions in China.

Overall, it is only recently that Chinese researchers have applied spatial approaches to explore the underlying reasons of population ageing at a local level. With a mixed conclusion, only a few studies were further carried out in northeast China. For example, Yang et al. (2016) [22] believed that the presence of state-owned economy and the implementation of family planning policies were the main determinants of population ageing in northeast China. These exogenous factors, to a certain degree, lead to an extremely low fertility rate, which in turn acts on the acceleration of population ageing. Wang et al. (2016) [24] mentioned the case of Yichun City in Heilongjiang province, arguing that in addition to demographic changes, emigration is the most important determinant of population ageing. As a large number of young labor force flowed out, inevitably there is an aggravation of population ageing on average. Even though these studies have integrated many social or economic perspectives in order to capture the multi-dimensional nature of ageing, limited progress has been made on understanding how the population ageing can lead to a deeper and more fundamental shift of the present economic and social orders, particularly for those resource-exhausted cities that lack economic growth points and industrial supports. With this in mind, the present study aims to better understand the root of ageing issues and the ageing trajectory in the context of northeast China. Based on the global and local spatial autocorrelation analysis, we initially describe the overall and partial spatial differences and evolutionary trends of population ageing. Then the spatial error model, the spatial lag model, and the geo-weighted regression model are respectively used for exploring the determinants of population ageing on average and across different regions. We expect the findings will bring more insights into the further improvement of regional ageing policies in China.

## 3. Research Questions and Methodology

### 3.1. Research Questions

Considering the gap in existing research and the social reality in the northeast region in China, the study proposes the following hypotheses:

**Hypothesis** **1.** *In northeast China, the ageing process of population is accelerating.*

**Hypothesis** **2.** *The ageing process of population is determined by multiple socio-economic factors.*

**Hypothesis** **3.** *Traditional demographic factors such as birth rate and mortality rate may not play a decisive role in determining the ageing situation in northeast China.*

In general, the hypothesized structure of relationships between the key variables allows us to explore the independent effects of population ageing theory-related variables. It also enables us to consider and compare the differences of these associations with population ageing over time and space, thus enriching the literature regarding the complexity nature of population ageing.

### 3.2. Data

The analysis is drawn from three major data sources: (1) the fifth national census statistics (2000), (2) the sixth national census statistics (2010), and (3) local statistics yearbooks. We believe that the period over 2000–2010 is of the highest importance in determining the current ageing situation in northeast China. The reason is twofold. On one hand, the national census data will be only released to the public every 10 years, thus the seventh wave is not accessible until 2020 and the fourth wave which was released in 1990 is not consistent with the fifth and sixth waves in terms of variable selection and definition. On the other hand, with the dramatic reform of state-owned enterprises since the late 1990s, the economic slowdown and the readjustment of social structure are especially evident in the northeast region. Therefore, the ageing pattern is more likely to be contemporarily formed during this period of time. According to the nomenclature of territorial units for statistics in China, the northeast region contains Liaoning province, Heilongjiang province, and Jilin province. At the secondary level, county-level data includes, in total, 194 spatial units (66 in Liaoning, 78 in Heilongjiang, and 50 in Jilin).

### 3.3. Models

#### 3.3.1. Spatial Autocorrelation (LISA) Statistics

We used both the global Moran statistics (GMS) and local Moran statistics (LMS) to describe (1) the overall spatial distribution of attribute values at the aggregate level; (2) the degree of spatial clustering/outlier of ageing population. If we define that n is the number of cities in the northeast region, X¯ is the averaged proportion of the aged population across all cities in the northeast region, $x_{i}$ and $x_{j}$ are the pixel intensity levels of the feature of interest which represent the proportion of aged population in city i and its neighborhood j, then the LMS $I_{i}$ for each city is expressed as:

(1) $$I_{i}=Z_{i}\sum_{j\neq i}^{n}W_{\mathrm{ij}}Z_{j}$$

where $z_{i}$ and $z_{j}$ are the deviations of $x_{i}$ and $x_{j}$ based on the mean intensity level X¯

(2) $$z_{i}=\frac{x_{i}-X¯}{s_{i}^{2}}$$

(3) $$\;z_{j}=\frac{x_{j}-X¯}{s_{j}^{2}}\;$$

(4) $$\;s_{i}^{2}=\frac{\sum_{j=1}^{n}{\left(x_{j}-X¯\right)}^{2}}{\left(n-1\right)}\;$$

Moreover, $W_{i,j}$ is the spatial weight, based on the standard of Queen Contiguity (for entities that share a common side or vertex), which implied the spatial interactions between cities $x_{i}$ and $x_{j}\;$ and $s_{i}^{2}$ is the standard deviation of $x_{j}$ next to ${\;x}_{i}\;$.

The following information can be obtained from LISA statistics shown above. First, averaging LMS $I_{i}$ gives the value of global Moran statistics (GMS) I:

(5) $$I=\frac{n}{S_{0}}\frac{\sum_{i=1}^{n}\sum_{j=1}^{n}w_{i,j}z_{i}z_{j}}{\sum_{i=1}^{n}z_{i}^{2}}$$

(6) $$\;S_{0}=\overset{n}{\underset{i=1}{\sum }}\overset{n}{\underset{j=1}{\sum }}w_{i,j}\;$$

The range of values of GMS is (−1, +1) and the range of values of LMS is (+$k_{1},-k_{2}$), where $k_{1}$ and $k_{2}$ are the upper and lower limits of LMS. Moran’s I was tested for significance using the Z test. Second, positive values of either LMS or GMS indicate a trend of clustering (a low margin between $x_{i}$ and $x_{j}$ in terms of the pixel intensity level) while negative values of those imply a trend of outlier (a wide margin between $x_{i}$ and $x_{j}$ in terms of the pixel intensity level). The larger magnitude of value| I | is, the stronger spatial autocorrelation it exhibits. Finally, clustered cities can be classified as HH (High–High) and LL (Low–Low) areas, where the population ageing of a certain city and its adjacent cities is higher/lower than that of the whole northeast region. Outliers can be classified as HL (High–Low) and LH (Low–High) areas, where the population ageing degree in a certain city is higher/lower than the overall regional average, and the population ageing degree in its adjacent cities is lower/higher than the overall regional average.

#### 3.3.2. SLM and SEM Estimations

Next, we explicitly model the proportion of population aged as the function of birth rate (live births per 1000 for one year), death rate (the number of deaths per 1000 for one year), illiteracy rate, the ratio of workers in health industries over the total employed population, the percentage of workers in the secondary industry over the total population employed, the ratio of urban population over the total population, the ratio of local residents (unmoved more than 6 months) to household population, then the basic econometric equation is shown as below:

(7) $$Y_{i}=\alpha +\beta_{1}\mathrm{birth}+\beta_{2}\mathrm{death}+\beta_{3}\mathrm{illiteracy}+\beta_{4}\mathrm{health}+\beta_{5}\sec\mathrm{ondary}+\beta_{6}\mathrm{urban}+\beta_{7}\mathrm{emigration}+\varepsilon_{i}$$

where Y is the proportion of the population aged in city i.

Considering the spatial dependence between independent variables and error terms, the simple application of OLS regression is likely to lead to a significant bias, thus the spatial lag model (SLM) and the space error model (SEM) model are introduced, respectively, for controlling the effects of spatially substantive dependence and spatial interaction. The SLM model is given as follows:

(8) $$Y=a+\rho \mathrm{WY}+\mathrm{bX}+\mu ,\mu \sim N\left(0,{\;\sigma }^{2}I\right)$$

where the vector of  W is the spatial matrix, ρ is the spatial autoregressive parameters, X is the n × k independent variable matrix (n is the number of cities, k is the number of independent variable), and μ is the random error term. From a methodological point of view, the application of SLM takes the effect of spatial structure within the northeast region into account, in addition to the independent effects of the spatial units (i.e., city). In comparison, the SEM model assumes the effect of stochastic disturbances on a particular city and that such an effect can be passed on to adjacent areas through the spatial structure. Its expression can be demonstrated as below:

Y=a+bX+ε

(9) $$\varepsilon =\lambda W\varepsilon +\mu ,\;\mu \sim N\left(0,{\;\sigma }^{2}I\right)$$

where the error term ε is further decomposed into two parts:(1) λWε i.e., stochastic disturbances and (2) μ i.e., the moving average of random error.  λ is the spatial error coefficient and the meanings of other parameters are the same as those in Equation (7).

#### 3.3.3. Geographical Weighted Regression (GWR) Estimation

The GWR method is often adopted when the relationship between dependent and independent variables is assumed non-stationary over space instead of constant on average. As a result, this method is also used for checking the robustness of the results generated from SLM and SEM. We expect an exploration on the variation of coefficients within an analytical framework can bring more insights into the causes of population ageing in the northeast region concerning a spatial–temporal disparity.

$$y_{i}=\beta_{0}\left(u_{i},v_{i}\right)+\underset{k}{\sum }\beta_{k}\left(u_{i},v_{i}\right)x_{\mathrm{ik}}+\varepsilon_{i}$$

(10) $$\hat{\beta }\left(u_{i},v_{i}\right)={\left(X^{T}W\left(u_{i},v_{i}\right)X\right)}^{-1}X^{T}W\left(u_{i},v_{i}\right)Y$$

As Equation (10) shows above, ${\;\beta }_{k}\left(u_{i},v_{i}\right)$ is the local regression coefficient of city i, $\beta_{0}\left(u_{i},v_{i}\right)$ is the parameter of intercept at city i, and $\varepsilon_{i}$ is the Gaussian error at city i. ${\;x}_{\mathrm{ik}}$ is the vector of independent variables, which can be also identified at city i. In contrast to an OLS setup, the GWR coefficient values of variables are spatially dependent.

### 3.4. Variables Measures

We propose the following demographic and socio-economic indicators as shown in Table 1:

## 4. Empirical Analysis

### 4.1. Spatial Demographic Analysis

**Figure 2:**
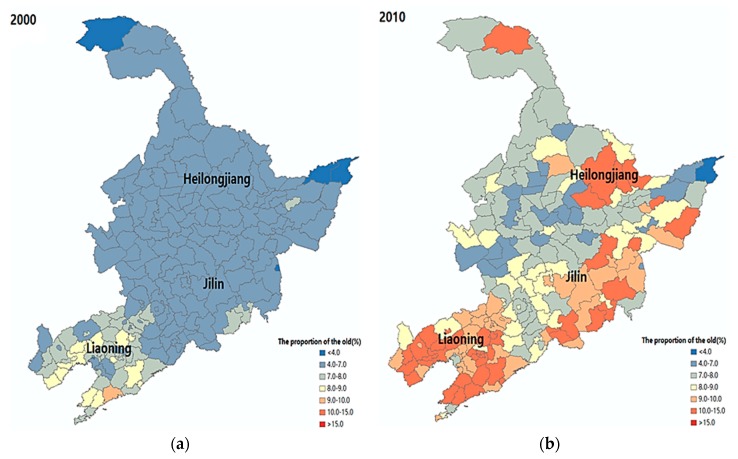
Ageing index of the cities in northeast China. (**a**) Year 2000; (**b**) Year 2010.

The main goal of this section is to evaluate the spatial impact of ageing in the northeast region. It is informative to outline the overall level of the aged population before considering its variation and the relationship with other socio-economic factors over time. Therefore, Figure 2 depicts the regional distribution of the aged population in the reference years of 2000 and 2010. Here, each pixel location i is given different colors according to its proportion of aged population over a total local population. By looking at Figure 2a, there is an indication that the share of the aged population significantly increased for many regions of northeast China over the period 2000–2010. In 2000, the proportion of the aged population in most regions of Heilongjiang and Jilin was only inside a range of 4–7%. In comparison, Liaoning province had a higher level of population ageing, as approximately 7–9% population was equal or greater than age 65. However, a quite different pattern can be captured in 2010. Figure 2b shows that in the most populated and developed regions of all three provinces, the proportion of aged population had significantly increased to 9–15%. These findings reveal that the population structure of the entire northeast region has undergone a dramatic ageing process in recent decades, particularly in large cities such as Shenyang, Dalian, and Harbin.

**Figure 3:**
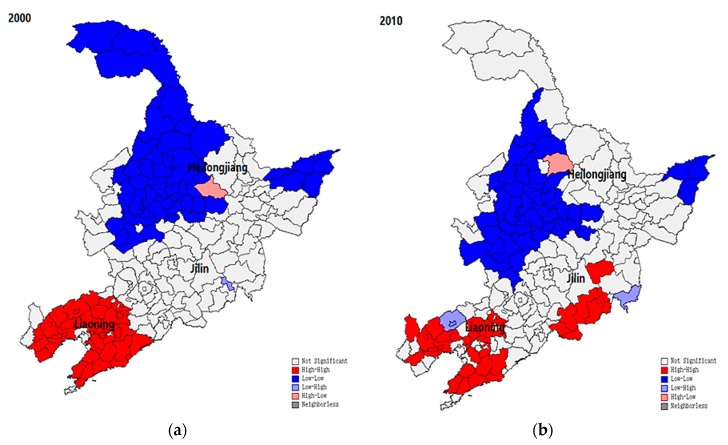
LMS index of the cities in northeast China. (**a**) Year 2000; (**b**) Year 2010.

Next, as described above, the global Moran statistics (GMS) and local Moran statistics (LMS) are adopted with different perspectives to deliver robust results. The values of GMS were 0.72 and 0.54 in 2000 and 2010, respectively; all reached the level of significance. This finding hints that in general, the spatial autocorrelation effect of population ageing was observed. The LMS analysis further reveals a significant variation of such an effect among different regions. As shown in Figure 3a,b, a clear clustering pattern of population ageing, no matter if the feature is ‘HH’ or ‘LL’, can be observed. In 2000, the ‘LL’ cities, where the proportion of ageing population is lower than that of the regional average (i.e., the zone with a comparatively slower ageing process), mainly concentrated in Heilongjiang province, whereas the ‘HH’ cities, where the proportion of ageing population is higher than that of the regional average (i.e., the zone with a comparatively faster ageing process), can be only observed in Liaoning province. In comparison, the size of the ‘LL’ region significantly decreased, and in addition to the existing ‘HH’ region in Liaoning, a number of cities in Jilin also exhibited a trend of clustering in 2010. These findings may prove the interdependency between population and urbanization. As the economic and political center of the northeast region, Liaoning province provides the highest supply of jobs in the heavy and manufacturing industries, while other regions such as Heilongjiang only have a limited effect. Therefore, it is not surprising to see that the ageing process was faster in the populated area. However, the ageing situation has drastically changed within one decade. Now, even the traditionally less populated areas with a lower level of economic development also experienced an increase in ageing population.

In a nutshell, the evolution of the ageing pattern is evident in the northeast region. Besides the various trends based on the detailed breakdown of data, there is a general expansion trajectory of ageing population, thus hypothesis 1 can be confirmed. This substantial change is very likely to be the consequence of socio-economic development and intensive out-migration of labor forces. Hence, an econometric discussion of the effects of these factors on this ageing situation will follow.

### 4.2. Econometric Analysis: OLS, SLM, and SEM

It is worth mentioning that the spatial models are used to (partially) deal with endogeneity issues caused by spatial autocorrelation. Tackling other endogeneity issues is difficult in this context due to the lack of good instruments and the nature of data, so the econometric results are interpreted as associations, which could further shed some light on identifying the possible casual links between socio-economic factors listed in Table 1 and population ageing. This is also the reason why a series of tests based on different model specifications are conducted in the next section for checking the robustness of our results.

Table 2 presents the econometric results using the census data in years 2000 and 2010. It appears that there is a significant difference among three model specifications. For example, the coefficient of illiteracy was not statistically significant in 2000 based on the OLS estimation, whereas that of 2010 was 1.41 based on the SEM estimation, with the level of significance. These findings suggest a model misspecification, most likely due to the large spatial heterogeneity across observations. The further residual analysis confirms the presence of spatial correlation, as the values of Moran’s I (error) were 4.16 and 6.11 for years 2000 and 2010, respectively; all reached the level of significance. Concerning the application of SLM and SEM, both the values of LM (Lag) and LM (error) were significant at 1% level. However, the LIK values of SEM in both years 2000 and 2010 were greater than those of SLM, while the AIC and SC values were also smaller. Therefore, we believe that the SEM model exhibits a higher level of goodness-of-fit. The results are shown as follows:

The birth rate had a significantly negative association with population ageing in the northeast region of China, indicating that a higher level of birth rate may improve the demographic structure, which in turn acts on mediating the process of population ageing. These results are consistent with previous studies, that is, population ageing is more likely to be observed in places with a lower birth rate. By contrast, the coefficients of mortality on the population ageing were 5.03 in 2000, whereas that in 2010 was 3.61, both reaching the level of significance. These findings imply that with the improvement of social health and economic development, a lower level of mortality is likely to restrain the population ageing process. The possible reason that may lead to this result is that the decrease of child mortality rate has a relatively greater impact. However, due to the data being not publicly accessible at the county level, we compared the mortality rates of the elderly population (i.e., age 65+) and the young population (i.e., 0–14 and 0–34), respectively, at the provincial level in order to capture such a pattern. As shown in Table 3, the mortality rate of the young population significantly decreased over the period 2000–2010, whereas that of the elderly population increased or decreased with a much lower rate. This trend was particularly observable in all three provinces in northeast China. This result, to a certain degree, supports the point of view from previous studies that the decrease of the morality rate of China’s young population is associated with a slowdown of population ageing, as the proportion of the absolute population aged above 65 eventually decreases with the presence of much more newborn babies and healthy teenagers [27,28].

Similarly, a significant positive association was observed between the illiteracy rate and population ageing in 2000 and 2010. As mentioned earlier, a lower level of illiteracy rate signals a better level of social provision, including education quality, thus attracting a working age population and eventually making the demographic structure younger. Notably, there was a positive association between health conditions and population ageing in 2000, as the improvements in healthcare potentially prolong the life span of local residents, which in turn acts on accelerating the population ageing process. However, such an association was not observable in 2010. The possible reason for this result is that the association between improving overall healthcare and extending the average life expectancy of local residents may vary at different stages of socio-economic development. In other words, improving basic health and medical care conditions in less-developed regions will, initially, significantly extend the average life expectancy of local residents (e.g., from age 30 to age 69), but as long as the average life expectancy has reached to a high level, a further improvement of basic healthcare would only cast a small effect on it (e.g., from age 70 to age 85).

It is generally well documented that factors such as birth rate, mortality, or healthcare conditions are important determinants of population ageing, but our results show that their associations with population ageing are significantly weakened over a decade. In comparison, the importance of industrial structure and out-immigration become higher. For example, the associations of the size of secondary sectors and urbanization with the population ageing were not significant in 2000, whereas their coefficients were 0.71 and 0.98, respectively, in 2010, with the level of significance. This positive association between the size of secondary sectors and population ageing reflects several realistic development difficulties in northeast China from a ‘structural’ perspective. On one hand, with the slow process of economic transition, the size of the traditional industries remains dominant over the last two decades, and even in many regions, its proportion statistically rises, as the local non-industrial economies continue to shrink. On the other hand, while the present industrial structure, to a great extent, remains unchanged, the demographic structure of the working population attached to it is also less likely to be altered by an inflow of new jobs, particularly those created in the tertiary sectors. Therefore, this is a different scope from measuring the output growth of local industries in general, as output growth decline is often negatively associated with the population ageing process. As long as the existing working population gets aged with no replacement, the unduly proportion of traditional industries accelerates the process of population ageing.

Similar to the industrial structure variable, the positive association between urbanization and population ageing was also significant. With the rapid development of urbanization, the local human settlement environment has been greatly updated, which in turn increases the longevity of the local population on average. It is particularly worth mentioning that the negative association between population mobility and population ageing became much stronger in 2010, compared to that of 2000, indicating that the population out-migration is likely to significantly deteriorate the ageing situation in the northeast region and such an association even appears to be larger than other factors, including birth rate, mortality, and urbanization. The northeast region had maintained a relatively young population due to the immigration of labor forces that arrived in the 1950s and 1960s, triggered by the national strategy of post-war economic development, but as mentioned earlier, an economic stagnation and the long process of industrial structure readjustment since the 1980s gave a boost to ageing that resulted in a high level of ageing population in 2010.

In summary, we suggest at least seven socio-economic factors that appear to influence the evolution of the ageing pattern in northeast China: (1) birth rate, (2) mortality, (3) education, (4) healthcare conditions, (5) the level of economic development, (6) urbanization, (7) population mobility. However, this ageing pattern was time-varying over the period 2000–2010. Birth rate, mortality, illiteracy, and healthcare conditions were associated with population ageing in 2000, which were further replaced by industrial structure, urbanization, and population mobility in 2010. Therefore, Hypotheses 2 and 3 can be confirmed.

### 4.3. Robustness Test

#### 4.3.1. Testing Alternative Variables

To our best knowledge, this study is one of few studies that investigates the diversified causes of population ageing at the county level in a context of northeast China. As the national bureau of statistics of China only provides very limited access to its regional account, the variable definition and selection may have biases due to data constraints. First, as demonstrated previously, a lower level of illiteracy has a positive association with population. However, because the older generation also generally has lower educational levels (i.e., a higher level of illiteracy) compared to younger generations, including the variable of illiteracy may cause the issue of simultaneity. Second, we measured the quality of healthcare by the number of workers employed in the health and medical industry. This measure can be further replaced by alternative variables, such as the overall level of heath care conditions. Finally, many studies [29,30] claim that providing incentives for improving regional economic structures might lessen population ageing because emigration would be lower. Therefore, in addition to exploring the role of industrial structure within this population ageing process, it may be more intuitive to directly examine the effect of improving economic structure on the ageing population in order to check whether advanced economic structure (not only industrial structure) attracts a young labor force.

In addressing these issues, we further replaced the variables mentioned above for testing the robustness of the model’s specification. Specifically, based on the supplementary data from China urban statistical yearbook, China’s county (city) socio-economic statistics yearbook, and China health statistics yearbook, we generated the variables of education expenditure per capita, the number of hospital beds per capita and the proportion of employees working in tertiary sectors to replace the variables of illiteracy, health employees, and industrial sector size, respectively, and we expect our results are robust across different model specifications. As shown in Table 4 (based on SEM model), the coefficient of education expenditure was –0.13, with the level of significance at 2000, whereas this significant association with population ageing was not observed in 2010. Compared to the results based on the variable of illiteracy, a higher level of local education expenditure also appears to improve the local quality of social provision, as does illiteracy, thus attracting more young labor to work and live at this place, and eventually slowing down the population ageing process. Such an association with population ageing is gradually weakening over time.

There is no accessible data regarding health public expenditure or health public professionals at the county level in northeast China. Instead, we used the variable ‘the number of hospital beds per capita’ as a proxy, as it is common practice for a local government to increase the number of hospital beds and associated healthcare expenditure to meet increasing demands; the higher density level of hospital beds a region would have, the higher level of healthcare services it would offer. Similarly, the results revealed a pattern that the positive association between healthcare and the population ageing process decreased over the period 2000–2010.

The last change made in this robustness check is that we used the variable of the proportion of employees working in tertiary sectors to test if the population ageing process was likely to be associated with economic structure changes. We first introduced the variable of tertiary sector size alone. As shown in column 4 and 8, in neither 2000 nor 2010 was the association between the size of tertiary sector and population ageing observed. The further estimations introducing both the size of secondary sectors and the size of tertiary sectors, as shown in column 5 and 9, suggest that there were no associations between them and population ageing in 2000; however, there was a positive association between the size of secondary sectors and population ageing in 2010, and the association between the size of tertiary sectors and population ageing was significantly negative. These findings can be deemed realistic. First, even though the variable of the proportion of employees working in tertiary sectors is ideal proxy representing the level of economic structure improvement, as mentioned earlier, northeast China is struggling with its economic transition, thus the demographic structure of the labor force is still mainly determined by the density (size) of the secondary industry, which most young labourers work in [31]. This appears to be the reason for why the association between the size of tertiary sectors and population ageing was not significant when it was introduced into the regression alone; second, in the case where both variables were introduced, the positive association between the size of secondary industries and population ageing in 2010 confirms that our result presented in Table 2 is robust. It would be interesting if a negative association between the size of tertiary sectors and population ageing was also observed. However, this result would only imply that its association with population ageing was very weak, which can be only exposed after controlling for the strong statistic effect of the size of secondary sectors.

In summary, compared to the results based on Table 2, it is believed that our results are highly robust. The sign or significance of these variables vary based on different perspectives, but we can still reach similar conclusions; that (1) there are substantial associations between the level of education (or the quality of healthcare) and the population ageing process in northeast China, but these associations were weakening over the period 2000–2010; (2) in this case where a region is struggling with economic transition, the spatial distribution of traditional industries may have a stronger explanatory power on explaining population ageing. These findings are also not contradictory to previous studies. For instance, Lei and Ye [32] showed that the level of education expenditure was negatively related to the level of population ageing in Jiangxi province; Nie and Yan [33] emphasized that there was no significant association between the size of tertiary sectors and population ageing in Fujian province. Yang et al. [22] found a positive relationship between the population ageing progress and the quality of healthcare in Jiangsu province.

#### 4.3.2. GWR Analysis

The SLM and SEM models provide a way of measuring the possible effects of a series of socio-economic factors in relation to population ageing over space but cannot take account variations between them. In this section, geographical weighted regression (GWR) was employed to gain greater insight into the differentiated impacts of these factors across cities in northeast China. The local coefficients of illiteracy rates and urbanization did not reach the level of significance in both 2000 and 2010, hence they were excluded.

**Figure 4:**
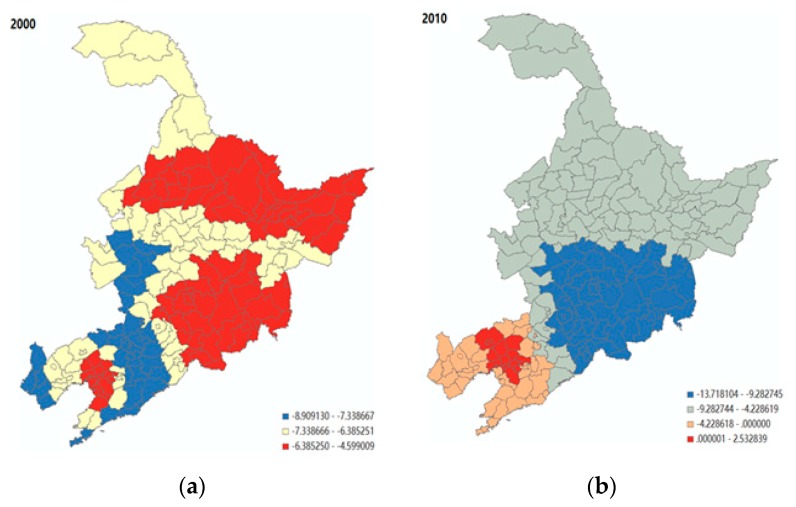
The spatial variability of the effect of birth rate on population ageing. Note: the coefficients within the Liaoning province were not significant in 2010. (**a**) Year 2000; (**b**) Year 2010.

**Figure 5:**
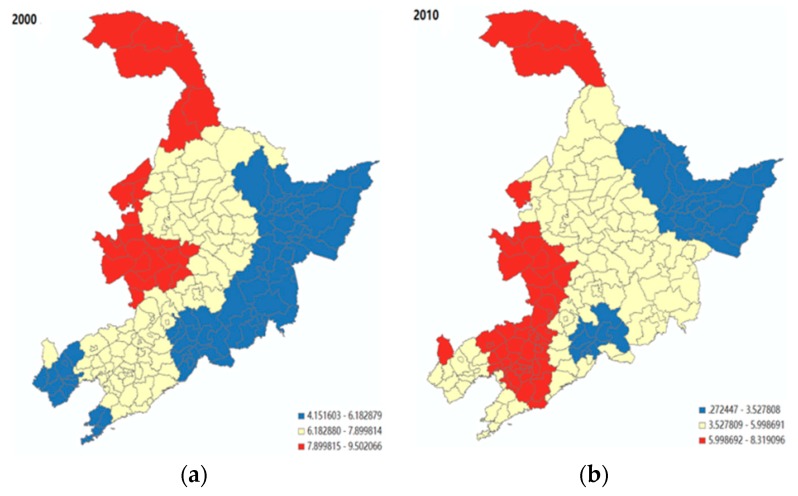
The spatial variability of the effect of mortality on population ageing. (**a**) Year 2000; (**b**) Year 2010.

**Figure 6:**
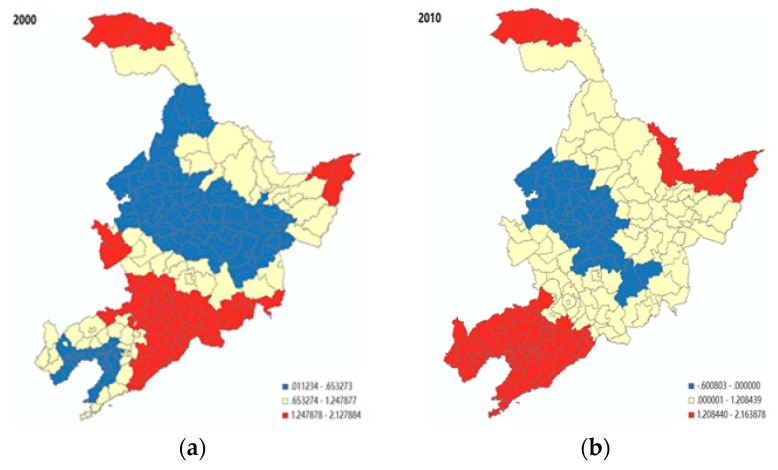
The spatial variability of the effect of industrial structure on population ageing. (**a**) Year 2000; (**b**) Year 2010.

Comparing the results shown in Figure 4, it is evident that the birth rate appears to have a significantly negative association with population ageing in almost all of the cities in 2000, with the level of significance. However, such a negative association between birth rate and population ageing was no longer significant for most of the cities in Liaoning province in 2010, where the socio-economic development was comparatively advanced (i.e., the red and orange parts in Figure 4b). For less-developed regions, such as Jinlin and Heilongjiang, the birth rate still played a role in influencing the population ageing. Next, as shown in Figure 5, there was a positive association between mortality rate and population ageing. In 2010, the size of this association was generally decreased, but with a higher level of spatial variation. Figure 6 shows that the level of economic development (i.e., the number of workers employed in the secondary industry, including mining, manufacturing, production and supply of electricity, gas, water, and construction, divided by the total population employed) had a significantly positive association with the population ageing in Jinlin, northeastern Liaoning, and northern Heilongjiang, compared to the rest of the regions in 2000 (i.e., the red part). However, the area with a significant positive influence on population ageing gradually shifted from Jilin to Liaoning, and to the northwest of Heilongjiang province in 2010 (i.e., the red part).

**Figure 7:**
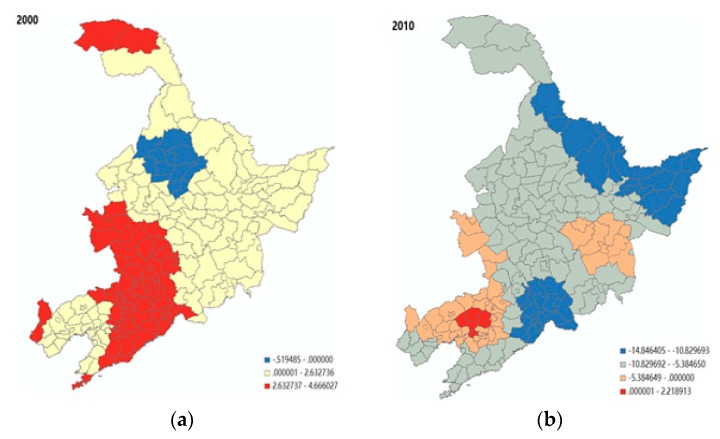
The spatial variability of the effect of healthcare conditions and population mobility on population ageing. (**a**) Healthcare conditions in 2000; (**b**) Population mobility in 2010. Note: the coefficients of healthcare conditions were not significant for all cities in 2010; the coefficients of population mobility were significant in the northern border of Jilin, eastern Jilin, and eastern Liaoning.

As far as healthcare conditions are concerned, a clear spatial pattern can be portrayed, as shown in Figure 7a. In western Jilin and northeastern Liaoning (i.e., the red part), there was a significantly positive association between the local population ageing and the level of healthcare conditions, with a set of coefficients within the range of 2.6–4.7. However, this positive association can be no longer observed in 2010 for almost the entire northeast region. Finally, the spatial variation of the effect of population mobility (see Figure 7b) hints that the level of this negative impact was overall higher in the less-developed areas, including the northern border of Jilin, eastern Jilin, and eastern Liaoning (i.e., the blue part) in 2010.

In summary, although the GWR estimation showed a slightly different ageing pattern (i.e., the insignificant effect of illiteracy and urbanization) compared to the SEM estimation, the results are still highly robust across different model specifications. Our demonstration about the associations between population ageing and socio-economic factors is likely to reveal that the local effects of a number of factors that are well documented in the ageing literature, such as birth rate, mortality, or healthcare conditions, were playing less important roles in affecting the ageing situation in many regions of northeast China, whereas the leading effect of the slow economic development accompanied with a population outflow was emerging.

## 5. Discussion 

The results presented in this paper highlight the important reasons behind the demographic change and the evaluation of ageing patterns in northeast China. The strong association between population ageing and the socio-economic process is evident from a temporal–spatial perspective. These findings also provide useful insights for policy makers for planning and implementing regional and local population policies.

The ageing pattern, mainly perceived through changes of the percentage of population equal to or above 65 years old over the total population in 2000 and 2010, has shown a general expansive trend of population ageing through the entire study area. In Liaoning province, where the level of socio-economic development is comparatively higher, the ageing population was mainly located in central cities such as Shenyang and Dalian, and the surrounding areas. In Heilongjiang and Jilin, a higher degree of population ageing was observed in the less-developed areas that are remote from the main economic–traffic corridor, including Daxinganling, Yichun, Jixi, and Yanji. The GMS estimation suggests that ageing is not an isolated phenomenon, as the cities with a high level of ageing population exhibit spatial autocorrelation. The LMS estimation further reveals that the presence of HH area (i.e., a cluster of cities with a higher level of population ageing) and LL area (i.e., a cluster of cities with a lower level of population ageing) is quite visual in Liaoning and most of the parts in Heilongjiang, respectively, indicating that there has been a convergence in population ageing in these areas.

The above findings bring a complexity of understanding to the causes of population ageing in the northeast region. It appears that the feature of population ageing can be expressed in many different ways and it appears to be determined by the intensive interactions of multiple socio-economic factors. The following econometric analysis suggests at least seven socio-economic factors that influenced that evolution of the ageing pattern from the different perspectives at the aggregate level, including birth rate, mortality rate, education, healthcare conditions, the level of economic development, urbanization, and population mobility. Particularly, the associated effects of birth rate, mortality rate, healthcare conditions, the level of economic development, and population mobility on the ageing population are robust across multiple model specifications. 

## 6. Conclusions

Although detailed examination of each factor has been demonstrated in the above section, it is still important to highlight several crucial links between population ageing and these socio-economic factors. First, there are complicated and diversified factors which may be associated with the deteriorating population ageing at the local level in northeast China. Many regions, due to the significant disparity in economic structure and historical background, have quite different social progress trajectories. Under such a situation, the cause of population ageing acceleration cannot be simply attributed to the change of a single factor. Second, the indicators that represent the minimal living standards, such as birth rate, mortality rate, education, or healthcare conditions, appear to play less decisive roles in affecting population ageing. In comparison, the issue of population ageing is more intuitively linked with the realistic need of the socio-economic development, for example, the urban-based industrialization. Finally, a strong association between migration outflow and population ageing is notably evident. This implies that migration outflow may have become one of the major forces that has caused a higher level of population ageing in many of the cities in northeast China.

Our findings reveal several theoretical and managerial implications. However, the major contribution of this study is to develop a more comprehensive framework that may explain the diversified causes of population ageing in a particular region in China. Thus, this study:

(1) Investigates the socio-economic dynamics of the population ageing at the disaggregate level in China. Our dataset contains a lower level of data hierarchy down to the county level. Such a composition allows us to more precisely understand the economic, social, and developmental problems caused by ageing.
(2) Develops a novel measure (i.e., the ratio of resident population to household population) to explore the role of population outflow within the population ageing process in addition to the traditional demographic or socio-economic factors.
(3) Utilizes several popular spatial models for highlighting the heterogeneous spillover effects of population ageing in a particular region.
(4) Provides meaningful references and materials for policy makers to better understand how specific ageing policies could be built up towards regions characterized as transitional economy.

Our findings have a range of managerial implications. At the aggregate level, the issues deeply rooted in the society, which are reflected in a spatial pattern of ageing, are likely to relate to the relatively backward socio-economic system. Therefore, improving the regional socio-economic ‘conditions’ in general may be more effective than directing support to a specific aspect. In other words, an ageing policy has to be generic and flexible according to the local socio-economic reality. At the local level, the findings of the paper show that several policy areas can be highly coordinated and provide a very detailed plan for policy makers to consider how the negative impact of population ageing can be efficiently alleviated. The complex nature of regional socio-economic systems means that the sustainable development of the northeast region may not be delivered by dichotomous policy interventions such as the control of birth rate or mortality rate as many of the previous studies argued [34,35]; instead, the implementation of an ageing policy shall be consistent and complementary with the principles of social benefits, for example, providing incentives for improving regional economic structures or by policies aimed at building up an adequate “tolerant culture” for slowing down the population outflow.

The main limitation of the paper is that other factors which are likely to be linked with the process of population ageing were not taken into consideration, namely public psychology, supply of jobs, social security, etc. However, the data to take into account these wider factors is not readily available at the disaggregate level. Also, this study elected to perform a cross-sectional analysis, thus a series of the associations proposed must be further verified with more rigorous methods. These limitations indicate two viable research directions: (1) use data collected from multiple sources to conduct a panel analysis to validate the present findings; (2) directly focus on the physical and mental health of the elderly in northeast China, in order to gain deeper insights into the function mechanism of ageing.

## Figures and Tables

**Table 1:** Definitions of variables.

Variable Labels	Description
Demographic factors	
Aged population	The local residents older than or equal to 65. Hence, the level of ageing is defined in the usual way as the number of aged population divided by the total population
Birth rate	The average annual number of births during a year per 1000 persons in the population
Mortality	The average annual number of deaths during a year per 1000 population
Socio-economic factors	
Illiteracy rate	The percentage of illiterate population over the total population
Health^1^	The number of employees who work in the health and medical industry divided by the total population employed
Industrial sector size	The number of workers employed in the secondary industry including mining, manufacturing, production and supply of electricity, gas and water and construction, divided by the total population employed
Urbanization level	The rate of urban population over the total population
Population mobility	The ratio of the total number of local residents who have unmoved more than 6 months over the total number of permanent residents

Note: (1) following Yuan et al. (2007) [25], we assume a lower level of illiteracy rate has a certain positive effect on the ageing of a population, as it signals a higher level of social provision including education quality; (2) following Yang et al. (2016) [22], we assume the decline of traditional industries is accompanied with a dramatic outflow of young skilled population, thus accelerating the process of population ageing in the northeast region; (3) following Liu et al. (2015) [26], a value of population mobility greater than 1 indicates a sign of population inflow whereas that of smaller than 1 implies an outflow. In less developed regions in China, the population flow mainly comprises of young and middle-aged labor, thus a population outflow inevitably increases the proportion of ageing population over total population and the ageing of population is likely to accelerate more quickly and severely.

**Table 2:** Spatial econometric estimation 1.

	Year 2000			Year 2010		
	OLS	SLM	SEM	OLS	SLM	SEM
Birth rate	−6.05 ***	−5.61 ***	−6.17 ***	−7.04 ***	−6.54 ***	−6.10 ***
	(−6.72)	(−4.67)	(−6.82)	(−6.87)	(−6.11)	(−7.31)
Mortality	8.33 ***	6.52 ***	5.03 ***	7.24 ***	5.75 ***	3.61 ***
	(13.99)	(11.34)	(12.03)	(10.00)	(10.47)	(11.02)
Illiteracy	0.22	0.88 ***	1.50 ***	1.01 **	1.03 ***	1.41 ***
	(0.16)	(5.83)	(3.76)	(2.43)	(3.61)	(3.73)
Health ^1^	3.77 ***	2.64 ***	0.94 **	1.07	0.60	0.31
	(5.37)	(4.87)	(2.40)	(0.06)	(0.10)	(0.14)
Industrial sector size	1.86 ***	1.16 ***	0.11	1.85 ***	1.13 ***	0.71 *
	(7.54)	(6.33)	(0.43)	(4.70)	(4.01)	(1.76)
Urbanization	−4.66 ***	−3.03 ***	−0.61	−0.68	0.11	0.98 **
	−8.52)	(−8.92)	(−1.06)	(−0.81)	(0.14)	(2.26)
Population mobility	5.12 ***	3.77 ***	0.03	−6.93 ***	−7.14 ***	−7.82 ***
	(3.89)	(3.41)	(0.12)	(-5.83)	(−4.36)	(−4.90)
constant	7.83 ***	5.03 ***	5.13 ***	7.03 ***	4.68 ***	7.85 ***
	(13.15)	(11.02)	(10.04)	(11.45)	(10.82)	(10.97)
LIK	−188.14	−160.26	−145.07	−265.63	−248.86	−242.28
AIC	392.29	338.53	306.15	547.27	515.73	500.56
SC	418.43	367.94	332.29	573.41	545.14	526.70
R^2^	0.73	0.80	0.86	0.63	0.70	0.74
Moran’s I (error)	4.16 ***			6.11 ***		
LM (lag)		47.22 ***			32.62 ***	
LM(error)			12.83 ***			30.57 ***
Obs	194	194	194	194	194	194

Note: dependent variable: the proportion of ageing population; T-values in parentheses, Sig: * *p* < 0.1, ** *p* < 0.05, *** *p* < 0.01; ‘Health ^1^’ is measured by the number of employees who work in the health and medical industry divided by the total population employed.

**Table 3:** Comparison of mortality rate between elderly population and young population.

	China		Liaoning		Jilin		Heilongjiang	
	2000	2010	2000	2010	2000	2010	2000	2010
0–14	0.043	0.011	0.016	0.004	0.020	0.003	0.013	0.003
65+	0.397	0.419	0.440	0.434	0.350	0.300	0.305	0.357
0–34	0.079	0.029	0.043	0.019	0.050	0.017	0.042	0.017

Note: the results are expressed in percentage points, unit: the ratio of deaths over the total population.

**Table 4:** Spatial econometric estimation 2.

	Year 2000				Year 2010			
	OLS	SLM	SEM^(1)^	SEM^(2)^	OLS	SLM	SEM^(1)^	SEM^(2)^
Birth rate	−2.61 ***	−2.48 ***	−3.08 ***	3.08 ***	−6.64 ***	−5.84 ***	−3.03 ***	−3.07 ***
	(−5.60)	(−6.97)	(−7.59)	(−7.58)	(−6.59)	(−6.73)	(−6.99)	(−6.46)
Mortality	7.48 ***	4.91 ***	4.97 ***	4.95 ***	7.56 ***	5.10 ***	4.59 ***	4.89 ***
	(12.96)	(9.63)	(9.20)	(9.07)	(9.21)	(6.72)	(5.22)	(5.69)
Education expenditure	−0.06	−0.12 **	−0.13 **	−0.13 **	−0.07	−0.11	0.01	−0.05
	(−0.77)	(−2.12)	(−2.40)	(−2.41)	(−0.46)	(−0.86)	(0.02)	(−0.33)
Health ^2^	0.35 *	0.41 ***	0.38 ***	0.38 ***	0.56 **	0.45 *	0.39	0.40
	(1.82)	(2.73)	(2.88)	(2.87)	(1.97)	(1.87)	(1.61)	(1.61)
Industrial sector size	2.10 ***	0.75 ***		0.10	2.20 ***	1.30 ***		1.25 ***
	(7.34)	(3.19)		(0.35)	(5.72)	(3.86)		(3.17)
Tertiary sector size	−0.36	–0.52	–0.58	–0.61	–1.86 *	–2.07 **	–1.26	–2.19 **
	(−0.54)	(–1.00)	(–1.13)	(–1.18)	(–1.95)	(–2.56)	(–1.58)	(–2.52)
Urbanization	–2.77 ***	–0.93 **	–0.61	–0.04	0.78	1.54 **	1.92 **	1.73 **
	(–5.16)	(–2.19)	(–1.06)	(–0.09)	(1.16)	(2.65)	(3.09)	(2.80)
Population mobility	5.36 ***	0.58	0.05	–1.00	–6.89 ***	–8.61 ***	–8.31 ***	–8.51 ***
	(3.07)	(0.42)	(0.12)	(–0.61)	(–3.61)	(–5.27)	(–4.40)	(–4.56)
constant	6.32 ***	2.57 ***	6.03 ***	6.11 ***	6.72 ***	3.83 **	8.64 ***	8.34 ***
	(7.52)	(3.54)	(8.74)	(8.31)	(4.48)	(2.84)	(6.41)	(6.23)
LIK	–200.00	–161.65	–224.76	–160.40	–266.44	–253.93	–258.21	–244.50
AIC	418.01	341.29	465.53	340.81	550.88	525.86	532.42	509.00
SC	447.42	373.49	491.68	370.71	580.29	555.27	558.56	541.68
R^2^	0.70	0.81	0.61	0.83	0.63	0.72	0.70	0.70
Moran’s I (error)	5.70 ***		10.12 ***		4.70 ***			
LM (lag)		79.19 ***				43.88 ***		
LM(error)				76.71 ***			48.01 ***	25.02 ***
Obs	194	194	194	194	194	194	194	194

Note: dependent variable: the proportion of ageing population; T-values in parentheses, Sig: * *p* < 0.1, ** *p* < 0.05, *** *p* < 0.01; ‘Health^2^’ is measured by the number of hospital beds per capita, SEM^(1)^ is the estimation excluding industrial sector size, and SEM^(2)^ includes both industrial sector size and tertiary sector size.
